# Work, Sex, and Substances: Differences in Self-Reported Chemsex Behaviors Between MSM Sex Workers and Digital Content Creators

**DOI:** 10.1007/s13178-026-01392-9

**Published:** 2026-07-22

**Authors:** Kristopher Jackson, Martha Whitfield, Starr Tomlinson, Glenn-Milo Santos

**Affiliations:** 1 College of Nursing & Health Sciences, University of Massachusetts Dartmouth, Dartmouth, MA, USA; 2 Center for AIDS Prevention Studies, University of California, San Francisco, San Francisco, CA, USA; 3 University of California San Francisco Medical Center, San Francisco, CA, USA; 4 Department of Community Health Systems, School of Nursing, University of California San Francisco, San Francisco, CA, USA

**Keywords:** Chemsex, Sex work, Men who have sex with men (MSM), Digital content creators, Sexualized drug use

## Abstract

**Background:**

Chemsex, the use of psychoactive substances to enhance sexual activity, may vary by occupational context. This study compared its prevalence, substance-specific patterns, and acquisition behaviors among two groups: male sex workers who have sex with men (MSM) and MSM who create sexually explicit digital content.

**Methods:**

Cross-sectional web-based survey of HIV-negative U.S. MSM (N = 769) with verified platform profiles. Group differences were assessed using chi-square tests with Benjamini–Hochberg correction, and independent associations were examined using multivariable logistic regression.

**Results:**

Sex workers reported higher prevalence of criminalized or controlled psychoactive substance use in occupational contexts than digital content creators (47.7% vs. 21.6%), with occupational group emerging as the strongest independent predictor of use (aOR = 3.36, 95% CI 2.41–4.69). Sex workers also reported greater use of poppers and erectile dysfunction medications more frequently obtained through unregulated acquisition channels.

**Conclusions:**

Occupational context shapes distinct chemsex use profiles among MSM, underscoring the need for occupation sensitive harm reduction and clinical outreach.

**Policy Implications:**

MSM sex workers and digital content creators represent occupationally distinct populations with divergent substance use profiles. Harm reduction, clinical screening, and outreach should be tailored to the risk environments and substances characteristic of each group, rather than treating all MSM engaged in sexual labor as a single, undifferentiated population.

## Introduction

Chemsex is defined as the intentional use of psychoactive substances to facilitate, enhance, or prolong sexual activity (Giorgetti et al., 2017; McCall et al., 2015; Stuart, 2019). Although the co-occurrence of substance use and sexual behavior has longstanding precedent, chemsex has increasingly been identified as a distinct public health concern, particularly among gay, bisexual, and other men who have sex with men (MSM) (Maxwell et al., 2019). Chemsex is associated with elevated rates of HIV transmission, sexually transmitted infections (STIs), psychological and/or physical dependence, and impaired consent negotiation (Amundsen et al., 2022; Pufall, 2018). Epidemiological data suggest that chemsex, sometimes referred to as “Party and Play” or “PNP,” is disproportionately prevalent among MSM. In the United States, rates of methamphetamine use are more than three times higher among gay and bisexual men compared to their heterosexual peers (Substance Abuse and Mental Health Services Administration, 2019). Beyond individual substance effects, chemsex prevalence among U.S. MSM has also been linked to the eroticization of risk, engagement in socially stigmatized behavior, and as a potential mechanism for managing internalized shame (Stuart, 2019).

## Common Chemsex Substances

Characterizing chemsex prevalence and associated harm across subgroups is complicated not only by gaps in the empirical literature, but also by the ambiguity in how chemsex is defined. There is no universally agreed-upon taxonomy of chemsex substances, and definitions of what constitutes chemsex in practice vary across geographic, demographic, clinical, and research contexts. This definitional variability has meaningful implications for prevalence estimates, risk assessment, and the comparability of findings across studies.

Globally, chemsex literature frequently centers on mephedrone, gamma hydroxybutyrate/gamma-butyrolactone (GHB/GBL), and crystal methamphetamine. While this core triad of substances has dominated recent literature in this area, empirical evidence suggests that substance use in sexualized contexts is not limited to these substances, particularly within occupational subgroups whose sexual behavior may differ from community MSM samples (Edmundson et al., 2018; Weatherburn et al., 2017). Some substances used by MSM during sex do not fit neatly into the chemsex category and may be taken alongside the substances that are most often associated with this behavior. These adjunctive substances include poppers (inhaled alkyl nitrites) and commonly prescribed erectile dysfunction drugs (see e.g. García-Pérez et al., 2022; Giorgetti et al., 2017; Pantalone et al., 2008). It is plausible that MSM sex workers and sexually explicit digital content creators, such as those producing content for platforms like OnlyFans or JustForFans, engage in substance use across a broader pharmacological spectrum (e.g., opioids, dissociatives, psychedelics, and prescription stimulants), given occupational demands, client expectations, and performance contexts distinct from recreational chemsex.

The present study therefore adopts a deliberately inclusive approach to substance use assessment, capturing both conventionally defined chemsex substances and additional substances reported in sexualized encounters. This approach is consistent with growing calls in the literature for more expansive and contextually sensitive frameworks for understanding sexualized substance use (Giorgetti et al., 2017; Stuart, 2019). An overview of the substances commonly associated with MSM chemsex and their physiological effects is presented in Table 1.

Studies from North America describe crystal methamphetamine as the primary stimulant used in sexualized drug contexts (Edmundson et al., 2018; Coronado-Muñoz et al., 2024). Among MSM engaging in chemsex, methamphetamine use is associated with prolonged sexual arousal, increased disinhibition, heightened confidence, or an elevated sense of intimacy (Graf et al., 2018; Weatherburn et al., 2017; Marshall & Werb, 2010; Li et al., 2021). Notably, methamphetamine is commonly used among MSM to prolong, sustain, and intensify sexual experiences (Maxwell et al., 2019), with some research reports describing methamphetamine-using MSM engaged in sexual activity for hours or days while under its influence (Semple et al., 2008).

Mephedrone, a synthetic cathinone more commonly encountered in European chemsex contexts, produces a similar profile of empathogenic and stimulant effects, including euphoria, heightened libido, increased sociability, and tactile sensitivity, though with a shorter duration of action that can lead to compulsive redosing (Measham et al., 2011; Palamar & Halkitis, 2006). GHB/GBL occupies a distinct pharmacological niche within the chemsex triad; users report euphoria, increased libido, disinhibition, and heightened sensory and sexual experience. Socially, GHB/GBL is often characterized as requiring ‘less commitment’ than other chemsex drugs, reflecting its comparatively short duration of action and mild after-effects at low doses. Notably, however, GHB/GBL has a very narrow therapeutic window that markedly increases overdose risk, particularly in combination with alcohol or other central nervous system depressants (Palamar & Halkitis, 2006; Brennan & Van Hout, 2014).

## Additional Adjunctive Substances

### Poppers (Alkyl Nitrites)

Alkyl nitrites, colloquially known as “poppers,” occupy a distinctive place in MSM sexual culture that predates contemporary chemsex by decades. First popularized in gay communities during the 1970 s and 1980 s, poppers became deeply embedded in the social and sexual fabric of those communities, and this cultural continuity persists today (Zmith, 2021). Their primary physiological effects include rapid smooth muscle relaxation and vasodilation, thereby facilitating receptive anal intercourse by reducing discomfort; the use of poppers is commonly associated with intensified sensation and sexual disinhibition (Schwartz et al., 2020).

Of particular clinical concern is the combination of poppers with PDE-5 inhibitors; both agents act via nitric oxide-mediated vasodilation, and their co-administration carries a risk of severe, potentially life-threatening hypotension (Smith & Romanelli, 2005). Poppers are not controlled substances in many global jurisdictions, although this regulatory landscape is evolving, with some countries moving toward tighter restrictions in recent years. Their widespread availability has nonetheless contributed to a broadly held perception of relative safety among consumers. Clinical risks include chemical burns (Chiang et al., 2024), methemoglobinemia (Barrangou-Poueys-Darlas et al., 2021; Sonck et al., 2022), and, with newer formulations containing isopropyl nitrite, poppers maculopathy (Bartolo et al., 2022). Data describing how poppers are integrated into commercial sexual encounters or digital content creation remain limited.

### PDE-5 Inhibitors

Phosphodiesterase type-5 (PDE-5) inhibitors (e.g., sildenafil, tadalafil, vardenafil) are widely used pharmaceutical agents in MSM sexual contexts (Park et al., 2021). Originally developed for the management of erectile dysfunction, these medications have been broadly adopted for recreational and occupational sexual enhancement, frequently outside formal medical supervision and via informal supply chains (Mostafa & Alghobary, 2022). In chemsex contexts, PDE-5 inhibitors are used alongside a wide range of non-prescribed substances, including MDMA and methamphetamine; among insertive partners, stimulants can impair erectile function, and PDE-5 inhibitors are commonly used to counteract this effect (Fisher et al., 2009). For digital content creators and sex workers, PDE-5 inhibitors may confer occupational advantages, and a recent survey of male adult entertainers found that more than two-thirds reported occupational use, rising to nearly 85% among the youngest performers (Dubin et al., 2018). Co-administration with nitrates or alkyl nitrites carries a risk of severe hypotension and syncope and represents an important safety consideration across all use contexts.

### Alprostadil

Alprostadil, another erectile dysfunction drug, is available in the U.S. as an intracavernosal injection. This medication is used to produce an erection even in the absence of sexual arousal. The injectable medication acts rapidly and is therefore most directly relevant to professional sexual contexts in which a reliable and sustained erection may be required. Its use has been documented in sex work and pornographic content production settings (Harker, 2025). Serious adverse effects include priapism, which requires urgent medical attention, as untreated cases may result in permanent erectile dysfunction. Repeated intracavernosal injection also carries risks of bruising, fibrosis, and infection, particularly when self-administered outside clinical supervision (Chew et al., 1997).

### Understudied Subgroups: Chemsex Among MSM Sex Workers and Digital Content Creators

As described above, epidemiological research has advanced our understanding of chemsex prevalence and harm profiles among MSM both in the United States and globally. Less attention, however, has been directed toward subgroups whose occupational contexts may shape both exposure and vulnerability in meaningful ways. More recently, chemsex has been examined as a marketed feature of internet-based sex work. A recent analysis of U.S. MSM sex worker advertisements found that sex workers presenting in hegemonically masculine ways were more likely to advertise chemsex as a service, and that advertising patterns varied by encounter type. Sex workers advertising more intimate, relational experiences, such as the “boyfriend experience,” were less likely to advertise chemsex to prospective clients (Jackson, 2024). These findings underscore the relevance of occupational context in shaping chemsex-related behavior, yet key subgroups within this broader landscape remain insufficiently examined.

MSM engaged in in-person commercial sex work and MSM who produce adult digital content represent two underexplored subgroups. The occupational contexts of each group may meaningfully shape their relationship with chemsex, including the perceived utility of substances such as methamphetamine to extend sexual performance (Drysdale et al., 2020) or to manage the emotional labor inherent to sex work (Barry et al., 2025; Benoit et al., 2015; Cano-Ruiz & Íñiguez-Rueda, 2026). Subscription-based online platforms such as OnlyFans and JustForFans have enabled a distinctive category of sexual labor mediated through content creation and audience management rather than direct in-person contact, the health and behavioral implications of which remain poorly characterized in the literature. In addition to the most “common” chemsex substances, data on the use of adjunctive sexual enhancement substances (e.g. poppers and erectile dysfunction medications) within these occupational subgroups are also limited.

### Study Purpose

The present study aims to characterize the chemsex patterns among MSM sex workers and digital adult content creators, including the substances used, frequency, and contexts of use. Although MSM sex workers and digital content creators may constitute overlapping populations in practice, treating them as a single, undifferentiated group risks obscuring meaningful differences in occupational context, risk exposure, and support needs. We examine each group separately with the aim of generating occupationally sensitive evidence to inform targeted harm reduction, support services, and the development of culturally competent clinical care frameworks responsive to the distinct identities, risk environments, and healthcare needs of MSM engaged in in-person sex work or digital content creation.

## Methods

This cross-sectional internet survey examined patterns of chemsex among HIV-negative MSM engaged in sex work, comparing two occupational subgroups: individuals conducting in-person commercial sexual encounters and those engaged exclusively in digital content creation. The chemsex data reported here were embedded within a broader cross-sectional survey examining sexual health and HIV prevention behaviors among MSM. An internet-based design was selected to maximize confidentiality and facilitate access to these hard-to-reach and stigmatized populations, including individuals who may not publicly identify as sex workers nor as MSM. To detect a 10-percentage-point difference in chemsex behaviors between groups, an a priori power analysis indicated a minimum sample of 356 participants per group (baseline prevalence = 30% [Jackson, 2024], α = 0.05, power = 0.80), a threshold the final sample met. All study procedures were approved by the University of California, San Francisco Institutional Review Board (Protocol #24–41890) prior to data collection.

### Study Design

The survey was administered using REDCap (Research Electronic Data Capture), a secure, HIPAA-compliant, web-based platform designed to support research data capture (Harris et al., 2009, 2019). To mitigate automated or inauthentic responding, a multi-layered bot detection protocol was implemented, incorporating Google reCAPTCHA verification at survey entry, strategically embedded honeypot items, and evaluation of completion times against established response-time thresholds (Leiner, 2019). Responses triggering any component of the protocol were flagged and excluded prior to analysis.

Prior to survey launch, the principal investigator collaborated with a community consultant and member of the MSM sex worker community to review the survey instrument. This community-informed review ensured items were appropriately worded for the target populations and helped identify potential gaps in the domains assessed.

### Participants and Eligibility Criteria

Eligible participants were required to belong to at least one of the two target populations: MSM sex workers or sexually explicit digital content creators. Inclusion criteria were as follows: (1) age 18 years or older; (2) current U.S. residence; (3) male sex assigned at birth; (4) endorsement of condomless anal intercourse with a male partner within the preceding six months in the context of digital content creation or an in-person commercial sexual encounter; and (5) an active, verifiable profile on a prespecified adult content or escort platfor^♦^ maintained for at least six months prior to enrollment. Participants provided their active platform username at survey completion to verify eligibility. Individuals meeting all criteria received a $50 honorarium.

### Sampling and Recruitment

Participants were recruited using a stratified sampling approach to enable both between-group and within-group comparisons of chemsex behaviors among digital and inperson MSM sex workers. Because individuals who engaged in digital or in-person sex work used platform-specific usernames that were verified at the time of survey completion, the likelihood of duplicate submissions to obtain multiple incentives was minimized. MSM sex workers and MSM digital content creators were recruited concurrently between September 2024 and October 2025 from geographically diverse U.S. regions using three complementary recruitment methods:

1. **Direct Contact**: Participants advertising in-person sex work services were contacted via publicly listed contact information associated with their profiles, including text message, email, or direct messaging. Digital content creators on platforms such as OnlyFans and JustForFans were recruited using the same approach.
2. **Digital Advertising**: Paid, targeted banner advertising was placed on MSM-oriented geosocial networking applications: Sniffies and Grindr.
3. **Chain Referral Sampling**: Participants who completed the survey were encouraged to share study information within their social and professional networks. Chain referral sampling is widely used to recruit hidden, stigmatized, or hard-to-reach populations, including people who use drugs (Biernacki & Waldorf, 1981).

### Informed Consent

Prospective participants were screened for eligibility using an electronic screening tool integrated within the REDCap platform. Those meeting all eligibility criteria were directed to a digital informed consent form describing the study purpose, procedures, potential risks and benefits, and the voluntary nature of participation. Participants provided electronic consent before proceeding to the survey. All participants completed the informed consent process and survey electronically on a personal device.

### Study Procedures and Study Variables

Following consent, participants completed a self-administered online survey. Sociodemographic data collected included age, race, ethnicity, educational attainment, sexual orientation, health insurance status, and zip code of primary residence.

Substance use in the context of work-related sexual encounters was assessed using a checklist of controlled or criminalized psychoactive substances used in the preceding six months. Given the absence of a universally accepted operational definition of chemsex, the substance list was intentionally broad and included: crystal methamphetamine, cocaine, prescription stimulants, ketamine, GHB/GBL, LSD, DMT, MDMA, heroin/opioids, and other. Additional, separate survey items addressed a broader range of adjunctive substances used in sexualized contexts, including poppers, PDE-5 inhibitors, and alprostadil, for which limited data exist in these occupational populations. Participants reporting ED medication use identified their acquisition sources from a structured list of options. Participants endorsing poppers use in an occupational context were similarly asked to identify the sexual contexts in which they used poppers, selecting all that applied from a structured list. All substance items, response options, and contextual prompts were reviewed by a sex worker community advisor to ensure face validity and culturally informed framing.

Sociodemographic associations were examined only for controlled or criminalized psychoactive substances. For the purposes of this analysis, occupational categories were treated as mutually exclusive; participants who reported engaging in both sex work and digital content creation were coded as sex workers, as their digital content creation and online presence were considered likely expressions of their broader occupational identity as sex workers. Data pertaining to HIV and STI prevention practices were collected as part of the parent study but are beyond the scope of the present manuscript and will be reported separately.

### Analytic Strategy

Descriptive statistics were calculated to characterize participant demographics and reported chemsex behaviors. Frequencies and proportions were computed for categorical variables. Bivariate associations between occupational subgroups and categorical outcomes were assessed using chisquare tests of independence. Where expected cell counts fell below five or observed zero cells were present, Fisher’s exact test was used in place of chi-square. Effect sizes for bivariate (2 × 2) comparisons were computed with the phi coefficient (φ; equivalent to Cramér’s V) and interpreted per Cohen (1988): small = 0.10, medium = 0.30, large = 0.50.

Where multiple simultaneous comparisons were conducted within a single outcome domain, the Benjamini-Hochberg (BH) procedure was applied to control the false discovery rate (FDR) at 5%, reducing the risk of Type I error inflation (Benjamini & Hochberg, 1995). The Benjamini-Hochberg procedure was selected for this analysis over more conservative familywise error rate corrections (e.g., Bonferroni) given the exploratory nature of the study, the moderate-to-large number of simultaneous comparisons conducted within each outcome domain, and the importance of maintaining adequate statistical power across subgroup analyses with relatively modest sample sizes. The BH procedure controls the expected proportion of false discoveries among significant results rather than the probability of any single false positive, representing a more appropriate balance between Type I and Type II error in the context of descriptive, hypothesis-generating research (Benjamini & Hochberg, 1995). Raw *p*-values and BH-adjusted *p*-values (*p_BH*) are reported for all multiple comparison analyses.

Multivariable logistic regression was used to examine sociodemographic predictors of substance use in the context of work-related sexual encounters, defined for the purposes of this analysis as the use of controlled or criminalized psychoactive substances. Covariates were selected a priori based on prior literature identifying demographic and behavioral correlates of chemsex among MSM, including age, race/ethnicity, educational attainment, relationship status, partner gender composition, and sexual orientation (see, e.g., Hammond et al., 2023). Where initial categorical specifications showed poor fit or unstable cell sizes, more parsimonious alternatives were evaluated using likelihood ratio tests and the Bayesian Information Criterion (BIC). Participants identifying as straight/heterosexual were excluded from multivariable regression analyses due to small cell sizes but were retained in descriptive analyses. Participants with missing data on key covariates were excluded using listwise deletion. All analyses were conducted using Stata SE v16.1 (StataCorp, College Station, TX), with statistical significance defined as *p* <.05.

## Results

**Fig. 1:**
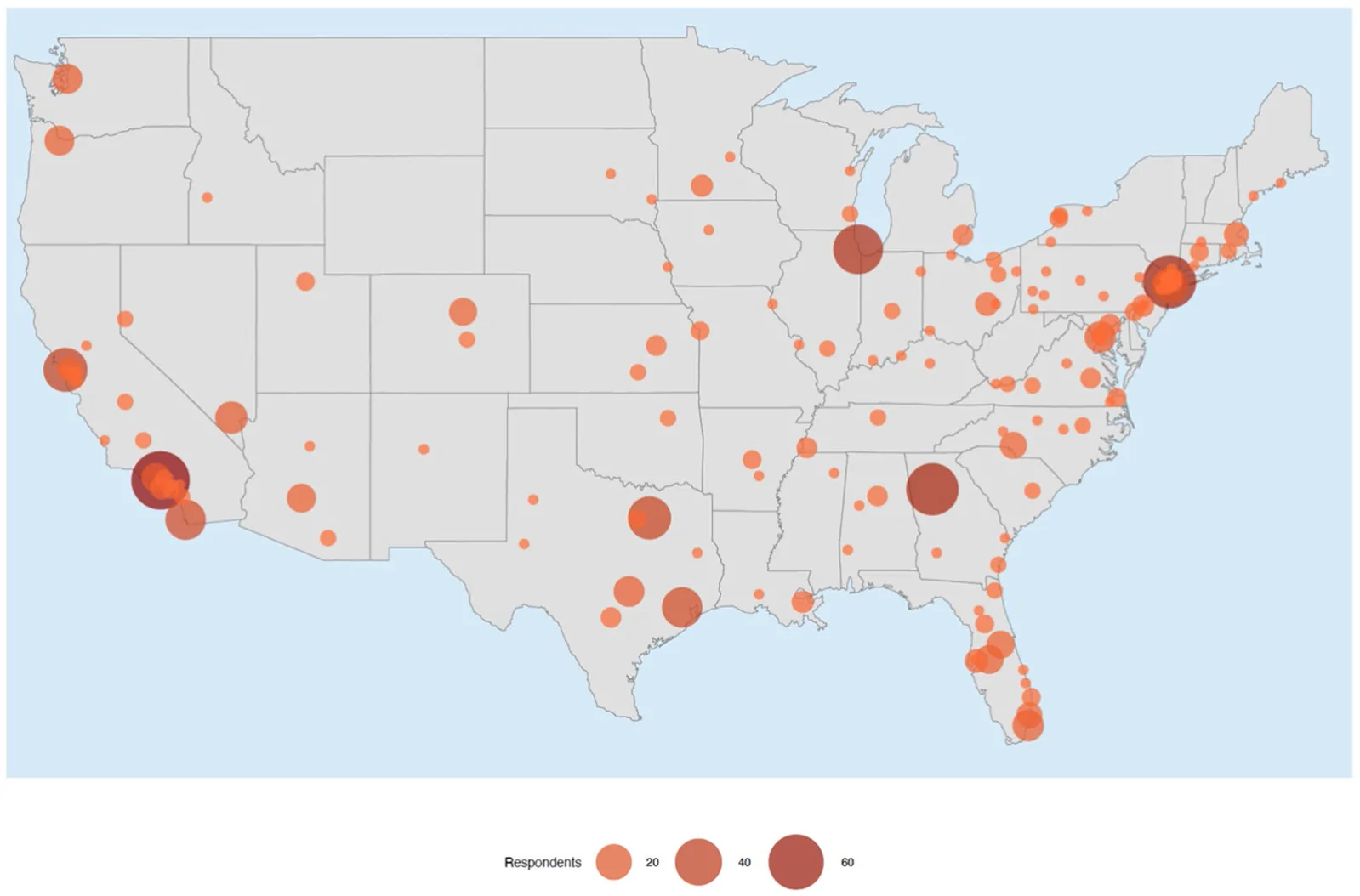
Geographic distribution of survey respondents by three-digit ZIP code prefix (*N*=769). Bubbles are plotted at USPS sectional center facility coordinates for each ZIP3 region; bubble size and color (orange to dark red) are proportional to respondent count, with larger, darker bubbles indicating higher concentrations. Map produced using the ggmap package (Kahle & Wickham, 2013). ***Note***: Alaska, Hawaii, and Puerto Rico are not pictured on this map; each with < 20 respondents

A total of 769 valid responses were retained for analysis following eligibility verification, data quality screening, and removal of automated or inauthentic submissions. The geographic distribution of respondents is shown in Fig. 1.

As highlighted in Table 2, the sample was predominantly non-Hispanic White (48.5%), followed by Hispanic or LatinX (22.1%) and non-Hispanic Black (17.3%) participants. The majority identified as gay or homosexual (71.9%), with smaller proportions identifying as bisexual (16.8%), pansexual (6.0%), and queer (3.3%). Educational attainment was relatively high, with 43.3% holding a bachelor’s degree or higher. The mean age was 32.8 years (SD = 8.3). The two occupational groups were largely comparable on all demographic characteristics assessed, including age, race/ethnicity, educational attainment, sexual orientation, and partner gender composition (all *p* >.05). Relationship status was the only exception. Digital content creators more likely to report being married (11.2% vs. 5.6%) and sex workers more likely to report being separated, divorced, or widowed (8.1% vs. 1.1%; χ^2^(4) = 31.54, *p* <.001).

### Patterns of Substances use

Table 3 presents chemsex prevalence and substance-specific use patterns across the two occupational subgroups. Sex workers were, overall, significantly more likely to report the occupational use of controlled or criminalized psychoactive substances than digital content creators (47.72% vs. 21.60%; χ^2^(1) = 57.62, *p* <.001, φ = 0.27), representing a medium-sized effect.

Among those endorsing occupational sexualized substance use, chi-square tests with Benjamini-Hochberg (BH) correction (FDR < 5%) revealed significant between-group differences for eight of ten substances assessed. Sex workers reported higher rates of methamphetamine, GHB/GBL, dimethyltryptamine (DMT), ketamine, prescription stimulants, and lysergic acid diethylamide (LSD) use (all *p_BH* < 0.05), with effect sizes ranging from small to medium (φ = 0.13 to 0.22). No significant differences were observed for cocaine or MDMA following correction, suggesting broadly comparable use of these substances across groups. Digital content creators reported significantly higher rates of heroin/opioid use (14.81% vs. 2.66%) and use of other substances (29.63% vs. 9.04%), both surviving BH correction (*p_BH* < 0.001) with small-to-medium effect sizes (φ = 0.23 to 0.26). Full statistical details are presented in Table 3.

Table 4 presents poppers use prevalence and context-specific patterns across the two occupational subgroups. Sex workers reported significantly higher rates of poppers use than digital content creators (70.05% vs. 57.33%; χ^2^(1) = [13.46], *p* <.001, φ = [0.13]).

Among poppers users, chi-square tests with BH correction (FDR < 5%) were conducted across nine contexts of use; one significant between-group difference was retained: sex workers were significantly more likely to report poppers use while giving analingus (46.01% vs. 33.02%, *p_BH* = 0.036, φ = 0.13), representing a small effect. No significant differences were observed across penetrative sexual contexts, including receptive anal intercourse, insertive anal intercourse, anal toy use, or masturbation (all *p_BH* > 0.05). Three further comparisons, receiving oral sex, giving oral sex, and receiving analingus, were significant prior to correction but did not survive adjustment (*p_BH* = 0.063, 0.095, and 0.069, respectively). The consistent directional pattern across oral-genital and oral-anal contexts, with sex workers reporting higher rates throughout, may warrant further investigation in larger samples. Full statistical details are presented in Table 4.

Table 5 presents PDE-5 inhibitor and alprostadil use prevalence and source patterns. Sex workers reported significantly higher rates of PDE-5 inhibitor use than digital content creators (69.04% vs. 47.73%; χ^2^(1) = [35.95], *p* <.001, φ = [0.22]). Among PDE-5 inhibitor users, two acquisition sources survived Benjamini-Hochberg correction: sex workers were more likely to obtain PDE-5 inhibitors through an internet pharmacy (29.04% vs. 17.88%, *p_BH* = 0.036, φ = 0.13) and through international purchase (14.34% vs. 6.70%, *p_BH* = 0.036, φ = 0.12), both representing small effects. No significant differences were observed for remaining acquisition sources following correction (all *p_BH* > 0.05).

Sex workers similarly reported significantly higher rates of alprostadil use than digital content creators (41.37% vs. 17.07%; χ^2^(1) = 54.55, *p* <.001, φ = 0.27), representing a medium-sized effect. Among alprostadil users, no acquisition source differences survived Benjamini-Hochberg correction (all *p_BH* > 0.05). Although sex workers reported higher rates of international purchase prior to correction (12.88% vs. 3.12%, *p* =.028), this did not survive adjustment (*p_BH* = 0.168). Full statistical details are presented in Table 5.

### Substance use: Multivariable Logistic Regression

To examine sociodemographic correlates of chemsex engagement, a multivariable binary logistic regression was conducted with chemsex participation (yes/no) as the outcome variable, defined as as the use of controlled or criminalized psychoactive substances in work-related sexual contexts, exclusive of poppers and erectile dysfunction medications. Relationship status was initially modeled using five categories; a likelihood ratio test indicated no significant improvement in fit over a binary partnered/unpartnered specification (LR χ^2^(3) = 6.29, *p* =.098), and the BIC favored the more parsimonious model (986.50 vs. 1018.08). Partner gender was collapsed from four categories to a binary variable (exclusively male and/or transgender partners vs. *any* female partners) and sexual orientation from six to three categories (gay, bisexual, and other sexual minority identities), each collapsed on the basis of likelihood ratio tests indicating no significant improvement in fit with more granular specifications. Straight/heterosexual-identifying participants (*n* = 11) were excluded from this model due to small cell sizes and theoretical distinctiveness from sexual minority subgroups but were retained in all descriptive analyses. Following additional exclusion of participants with missing data on race/ethnicity (*n* = 21) or relationship status (*n* = 8), 733 participants were included in the final model (LR χ^2^(15) = 80.92, *p* <.001). After adjusting for age, race/ethnicity, education, relationship status, partner gender, and sexual orientation, occupational group was the strongest predictor of chemsex, with sex workers having 3.36 times the odds of endorsing chemsex in the preceding 6 months as compared to digital content creators (aOR = 3.36, 95% CI 2.41–4.69, *p* <.001). Bisexual identity was negatively associated with chemsex engagement, with bisexual participants showing lower odds than gay-identified participants (aOR = 0.49, 95% CI 0.28–0.84, *p* =.010). Other sexual minority identities showed a directionally consistent but non-significant pattern (aOR = 0.57, 95% CI 0.31–1.05, *p* =.073). Being partnered was similarly associated with a non-significant reduction in odds of chemsex engagement (aOR = 0.67, 95% CI 0.43–1.02, *p* =.060). Full results are presented in Table 6.

## Discussion

This study examined chemsex engagement, substance use patterns, and the use of sexual enhancement substances among two understudied occupational subgroups of MSM: digital content creators and sex workers. To our knowledge, this is among the first studies to directly compare these groups on occupational sexualized substance use outcomes, and the findings reveal substantively meaningful differences that extend beyond overall prevalence to encompass substance-specific patterns, contexts of use, and modes of acquisition. Taken together, the results underscore the relevance of occupational context as a determinant of sexualized substance use risk among MSM.

### Sexualized Substance use and Occupational Context

The overall prevalence of chemsex engagement was substantially higher among sex workers than digital content creators (47.72% vs. 21.60%) and occupational group was the strongest independent predictor of chemsex engagement in the final multivariable model. Sex workers exhibited more than three times the adjusted odds of reporting chemsex compared to digital content creators (aOR = 3.36, 95% CI 2.41–4.69, *p* <.001). This finding is consistent with prior research documenting elevated substance use among MSM engaged in commercial sex work (Berg et al., 2019) and with research positioning chemsex as a socially and contextually embedded behavior shaped by the demands, norms, and social environments in which sexual encounters occur (Mundy et al., 2025). Notably, the self-reported prevalence of chemsex in this sample is considerably higher than the *advertised* prevalence of chemsex services documented in earlier studies of internet-based sex workers using data from the same advertising platform^♦^, which ranged from 22.4% to 28.5% (Jackson & Judge, 2021; Jackson, 2024). The persistence of this association after adjustment for demographic covariates suggests that the relationship between sex work and chemsex is more complex than sociodemographic covariates in our model and may instead reflect the realities of the work itself (e.g. performance demands, client expectations, and the social environments in which commercial sex encounters occur).

The prevalence of chemsex among digital content creators (21.60%), while significantly lower than among sex workers, is still noteworthy. This figure is broadly consistent with prevalence estimates reported among general MSM samples in prior epidemiological research (Georgiadis et al., 2025), suggesting that MSM digital content creators should not be treated as a low-risk group with respect to chemsex engagement and its associated harms. The distinct occupational context of digital content creation, which involves the performance of sex for a mediated audience rather than direct client contact, may generate its own configurations of sexualized substance use risk that differ qualitatively, rather than merely quantitatively, from those associated with in-person sex work.

### Substance-Specific Patterns

Among chemsex-reporting participants, chi-square tests with BH correction revealed significant between-group differences for eight of ten substances assessed. Sex workers reported significantly higher rates of several substances commonly associated with chemsex in the MSM literature, including methamphetamine, GHB/GBL, and ketamine, as well as substances less centrally featured in the chemsex literature, including prescription stimulants, DMT, and LSD. The elevated rates of methamphetamine and GHB/GBL use among sex workers are consistent with prior research documenting the instrumental use of substances to manage the physical and emotional demands of commercial sex work (Drysdale et al., 2020; Barry et al., 2025; Benoit et al., 2015).

### Opioid use

A notable finding in the substance use data is the significantly higher rate of opioid use in chemsex contexts among digital content creators as compared to sex workers (14.81% vs. 2.66%, *p_BH* < 0.001, φ = 0.26). This finding is counter-intuitive given the overall lower chemsex prevalence among digital content creators and warrants particular attention. Given that snowball sampling was among the methods used to recruit participants, it is possible that we tapped into a small subcommunity of opioid users in the content creator arm of this study, which may have resulted in overrepresentation in this sample. Opioid use in sexual contexts has been relatively underexplored in the chemsex literature, which has traditionally focused on stimulants and depressants perceived to have more desirable effects on sexual performance. It is also important to note that, because of the increasingly toxic and adulterated unregulated drug supply, the substances participants reported using may contain other contaminants or adulterants that were not captured by self-report, a consideration that has implications for interpretation and for harm-reduction messaging.

### Self-Reported Poppers use

Sex workers reported significantly higher overall rates of poppers use compared to digital content creators (70.05% vs. 57.33%). The contextual patterns of use, however, reveal a more nuanced picture. No between-group differences were observed in penetrative sexual contexts, including receptive and insertive anal intercourse, anal toy use, and masturbation. Following BH correction, one significant difference was retained: sex workers were significantly more likely to report poppers use while giving analingus (46.01% vs. 33.02%, *p_BH* =.036, φ = 0.13). Three further oral-genital and oral-anal comparisons were significant prior to correction but did not survive adjustment. The consistent directional pattern across these contexts, with sex workers reporting higher rates throughout, may reflect differences in the extent to which poppers are integrated into a wider range of sexual activities beyond those typically associated with the vasodilatory and muscle-relaxant effects of alkyl nitrites. For sex workers, the euphoric ‘rush’ produced by poppers may serve as a mechanism to generate passion or arousal in contexts where genuine desire may not exist, a consideration particularly relevant to commercial sexual encounters. Whether this pattern is driven by occupational norms, client preferences, or other contextual factors warrants further investigation.

### PDE-5 Inhibitor and Alprostadil use

Sex workers reported significantly higher rates of both PDE-5 inhibitor use (69.04% vs. 47.73%) and alprostadil use (41.37% vs. 17.07%) compared to digital content creators. The rates of PDE-5 inhibitor use in both groups are consistent with prior research documenting the widespread use of erectile dysfunction medications among MSM adult entertainers (Dubin et al., 2018). The rates of alprostadil use are particularly notable. Alprostadil is not typically encountered in recreational sexual contexts in the general MSM population, and its prevalence in the present sample suggests a degree of performance-oriented behavior not previously documented in the peer-reviewed literature on this topic or this population.

The acquisition source data introduce an additional dimension of clinical concern. Among PDE-5 inhibitor users, sex workers were significantly more likely to report purchasing medications abroad (*p_BH* = 0.036, φ = 0.12) and through internet pharmacies (*p_BH* = 0.036, φ = 0.13), both surviving BH correction. These patterns may reflect the documented mobility of MSM sex workers as a work-force (Jackson & Santos, 2023). For alprostadil (which was added to the survey based on input from a sex worker community advisor), no acquisition source differences survived correction, though sex workers showed higher rates of international purchase prior to adjustment (*p* =.028, *p_BH* = 0.168). These acquisition patterns raise important questions about medication quality, dosing accuracy, and the absence of clinical oversight, particularly given the established risks associated with PDE-5 inhibitor use in combination with substances like poppers. Notably, acquisition via a primary care provider did not differ significantly between groups for either medication. This suggests that a meaningful proportion of users in both groups are accessing these substances through formal clinical channels and mayrepresent a potential point of intervention for harm reduction and clinical education.

### Limitations

Several limitations of the present study warrant consideration. First, as noted in the Methods, participants were recruited through online platforms and social media as part of a broader cross-sectional survey examining HIV/STI prevention behaviors among HIV-negative MSM. This sampling approach may overrepresent digitally engaged individuals and may not be representative of the broader populations of MSM sex workers and digital content creators, including those living with HIV. Second, all data were self-reported and subject to social desirability bias, which may lead to underreporting of stigmatized behaviors, including sexualized substance use and the use of sexual enhancement substances.

The cross-sectional design of this study limits causal inference; the observed associations between occupational groups and chemsex outcomes cannot be interpreted as evidence that occupational context causes chemsex engagement, and unmeasured confounders may account for some portion of the observed differences. Further, the six-month recall period used to assess substance use does not permit meaningful inference about the co-occurrence of substance categories within individual sexual encounters; reporting overlap between substance categories at this level of temporal aggregation conveys no interpretable behavioral or contextual information about how, when, and/or why substances may be used together. Additionally, the respondents in this sample were geographically concentrated in large urban centers, which limits generalizability to MSM in non-urban settings. Notwithstanding these limitations, the involvement of a sex worker community advisor in the design and implementation of this study helped to mitigate several of the above concerns, particularly with respect to our recruitment messaging and culturally informed framing of survey items related to stigmatized behaviors.

### Implications and Future Directions

The findings of this study have several implications for clinical practice, harm reduction, and future research. The elevated prevalence of chemsex and sexual enhancement substance use across both occupational groups, situated within a broader MSM population already at elevated risk, suggests that MSM engaged in sex work and digital content production represent priority populations for targeted chemsex-related screening, harm reduction, and intervention. Notably, the occupationally specific substance use patterns observed within each group underscore the need for interventions that extend beyond generic MSM-focused chemsex programming. The distinct substance profiles observed between groups, and particularly the elevated opioid involvement among digital content creators, underscore the importance of moving beyond aggregate chemsex prevalence metrics to assess substance-specific risk patterns in occupationally defined subgroups. The widespread use of PDE-5 inhibitors and alprostadil, particularly through unregulated acquisition channels, represents an underappreciated harm reduction concern that should be incorporated into clinical and public health messaging directed at these populations. Given the higher prevalence of HIV among sex workers observed in this sample, there is a particular need to understand the patterns and contexts of chemsex engagement in this population, as sex workers living with HIV may face multiple intersecting stressors and layered stigma that shape both sexualized substance use practices and access to harm reduction services. In terms of policy, these findings highlight the need for harm reduction messaging and services that are tailored to the specific substance use profiles and occupational contexts of MSM sex workers and digital content creators. The concentration of digital content creators on subscription-based platforms presents a novel opportunity for platform-based outreach, including the integration of screening tools, peer-delivered counseling, and linkage to harm reduction and treatment services within digital environments already frequented by this population.

Future research should seek to characterize the motivational and contextual drivers of chemsex engagement within each of these two occupational groups using qualitative and mixed-methods approaches, with particular attention to the role of platform structures, audience dynamics, and occupational norms in shaping sexualized substance use practices among digital content creators. Longitudinal studies would be valuable in assessing the temporal relationship between occupational entry and chemsex initiation, and in evaluating the extent to which chemsex-related harms accumulate differentially across occupational contexts over time.

## Conclusion

Taken together, these findings suggest that where and how MSM engage in sexual labor shapes not only whether they engage in chemsex, but which substances they use and how they access them. Sex workers exhibited higher overall chemsex prevalence and reported greater use of methamphetamine, GHB/GBL, and performance-enhancing medications, including PDE-5 inhibitors and alprostadil, often procured through informal and unregulated channels. Digital content creators, despite lower overall chemsex prevalence, showed an unexpectedly elevated rate of opioid involvement in sexual contexts. Taken together, these divergent profiles suggest that aggregate chemsex prevalence metrics are insufficient for characterizing risk across occupationally distinct subgroups, and that intervention priorities may differ meaningfully between them.

These findings have direct implications for clinicians, harm reduction providers, and platform-based outreach programs. These groups should consider occupationally tailored approaches to screening, counseling, and linkage to care. The widespread acquisition of prescription medications through unregulated and international sources represents an under-appreciated and under-addressed clinical concern.

## Figures and Tables

**Table 1:** Commonly used chemsex substances

Substance	Route(s) of Administration	Intended/Reported Effects
Cocaine (2024)	Snorted, smoked, injected, rectal (e.g. ‘booty-bumped’). (San Francisco AIDS Foundation (n.d.)	Euphoria, elevated energy, increased talkativeness, and heightened mental alertness; appetite and sleep suppressant
Methamphetamine (2024)	Snorted, smoked, injected (slamming) (San Francisco AIDS Foundation 2018), oral, rectal (e.g. booty-bumped’) (San Francisco AIDS Foundation (n.d.)	Heightened sexual arousal, prolonged wakefulness, disinhibition, increased energy
Mephedrone (2025)	Snorted, oral, injection (less common)	Euphoria, empathogenesis, increased libido, heightened tactile sensation, sociability
GHB/GBL (2025)	Oral	Euphoria, disinhibition, heightened sensory perception, muscle relaxation Note: Although GBL converts to GHB once metabolized, its superior absorption results in greater bioavailability relative to an equivalent dose of GHB.
Ketamine (2024)	Snorted, injection, oral	Dissociation, disinhibition, reduced pain perception, euphoria at sub-anesthetic doses
MDMA (2024)	Oral, snorted	Empathogenesis, heightened tactile sensation/sensitivity, emotional closeness, increased libido

**Table 2:** Sample characteristics by occupational group (*N* = 769)

	Digital Content Creators (*n* = 375)		Sex Workers (*n* = 394)		Entire Sample (*N* = 769)		*p*
	M	SD	M	SD	M	SD	
**Age**	32.5	8.4	33.1	8.3	32.8	8.3	0.275
	Digital Content Creators (*n* = 375)		Sex Workers (*n* = 394)		Entire Sample (*N* = 769)		*p*
	n	%	n	%	n	%	
**Race/Ethnicity** ^a^							0.074
Non-Hispanic White	177	47.20%	196	49.70%	373	48.50%	
Hispanic or LatinX	75	20.00%	95	24.10%	170	22.10%	
Black or African American	71	18.90%	62	15.70%	133	17.30%	
Asian	15	4.00%	6	1.50%	21	2.70%	
American Indian or Alaska Native	3	0.80%	0	0.00%	3	0.40%	
More than one race	23	6.10%	19	4.80%	42	5.50%	
Other	2	0.50%	4	1.00%	6	0.80%	
**Education**							0.357
High school or less	85	22.70%	71	18.00%	156	20.30%	
Some college or associate’s degree	129	34.40%	138	35.00%	267	34.70%	
Bachelor’s degree	116	30.90%	127	32.20%	243	31.60%	
Graduate or professional degree	45	12.00%	58	14.70%	103	13.40%	
**Sexual Orientation**							0.283
Gay	277	73.90%	276	70.10%	553	71.90%	
Bisexual	54	14.40%	75	19.00%	129	16.80%	
Heterosexual/Straight	3	0.80%	8	2.00%	11	1.40%	
Pansexual	26	6.90%	20	5.10%	46	6.00%	
Queer	12	3.20%	13	3.30%	25	3.30%	
Other	3	0.80%	2	0.50%	5	0.70%	
**Relationship Status** ^b^							<0.001***
Single	217	57.90%	205	52.00%	422	54.90%	
Casually Dating	54	14.40%	75	19.00%	129	16.80%	
Committed Relationship or Engaged	56	14.90%	54	13.70%	110	14.30%	
Married	42	11.20%	22	5.60%	64	8.30%	
Separated/Divorced/Widowed	4	1.10%	32	8.10%	36	4.70%	
**Partner Gender (Last 6 Months)**							0.09
Exclusively men and/or transgender partners	289	77.10%	280	71.10%	569	74.00%	
Mostly men and/or transgender partners	51	13.60%	80	20.30%	131	17.00%	
Equal numbers of men, women, and/or transgender partners	26	6.90%	23	5.80%	49	6.40%	
Mostly women, some men and/or transgender partners	9	2.40%	11	2.80%	20	2.60%	

Percentages may not sum to 100% due to rounding. Age compared using independent samples t-test; categorical variables compared using Pearson chi-square tests.

a. Missing/prefer not to respond (*n* = 21) excluded from chi-square analysis; *n* = 748 for race/ethnicity comparison.

b. Missing/prefer not to respond (*n* = 8) excluded from chi-square analysis; *n* = 761 for relationship status comparison.

* *p*<.05;

** *p*<.01;

*** *p*<.001

**Table 3:** Chemsex engagement by occupational group and substances used (*N* = 769)

Chemsex	Digital Content Creators (*n*=375)	Sex Workers (*n*= 394)	χ^2^(1)	*P*	*φ*	
Yes	81 (21.60%)	188 (47.72%)				
No	294 (78.40%)	206 (52.28%)				
*Total*	375	394	57.62	<0.000	0.27	
Chemsex Substance(s) Used	Digital Content Creators (**n** =81)	Sex Workers (**n**=188)		*p*	*p_BH*	*φ*
Methamphetamine	28 (34.57%)	109 (57.98%)	12.42	<0.000	0.001*	0.22
Cocaine	22 (27.16%)	68 (36.17%)	2.06	0.151	0.168	0.09
Prescription Stimulants	3 (3.70%)	27 (14.36%)	6.49	0.011	0.015*	0.16
Ketamine	17 (20.99%)	71 (37.77%)	7.24	0.007	0.012*	0.16
GHB/GBL	30 (37.04%)	111 (59.04%)	10.99	0.001	0.002*	0.20
LSD	1 (1.23%)	15 (7.98%)	4.60	0.032	0.040*	0.13
DMT	5 (6.17%)	47 (25.00%)	12.87	<0.000	0.001*	0.22
MDMA	21 (25.93%)	52 (27.66%)	0.09	0.769	0.764	0.02
Heroin/Opioids	12 (14.81%)	5 (2.66%)	14.13	<0.000	<0.001*	−0.23‡
Other	24 (29.63%)	17 (9.04%)	18.57	<0.000	<0.001*	−0.26‡

* Significant after Benjamini–Hochberg correction (FDR < 0.05)

‡ Negative φ values reflect the direction of the association; absolute values indicate effect size magnitude

**Table 4:** Poppers use by occupational group and sexual contexts (*N* = 769)

Poppers Use	Digital Content Creators (*n* =375)	Sex Workers (*n* =394)	χ^2^(1)	*P*	*φ*	
Yes	215 (57.33%)	276 (70.05%)				
No	160 (42.67%)	118 (29.95%)				
*Total*	375	394	13.46	<0.000	0.13	
Context of Poppers Use	Digital Content Creators (*n*=215)	Sex Workers (*n*=276)	χ^2^(1)	*p*	*p_BH*	*φ*
Receptive anal intercourse (bottoming)	154 (71.63%)	202 (73.19%)	0.15	0.701	0.818	0.02
Insertive anal intercourse (topping)	131 (60.93%)	173 (62.68%)	0.16	0.692	0.818	0.02
Anal toy use	112 (52.09%)	141 (51.09%)	0.05	0.825	0.825	−0.01
Receiving oral sex	99 (46.05%)	158 (57.25%)	6.08	0.014	0.063	0.11
Giving oral sex	90 (41.86%)	141 (51.09%)	4.13	0.042	0.095	0.09
Receiving analingus (e.g. rimming)	80 (37.21%)	131 (47.46%)	5.19	0.023	0.069	0.10
Giving analingus	71 (33.02%)	127 (46.01%)	8.48	0.004	0.036*	0.13
Masturbation	128 (59.53%)	160 (57.97%)	0.13	0.727	0.818	0.02
Other	9 (4.19%)	16 (5.80%)	0.65	0.420	0.756	0.04

* Significant after Benjamini–Hochberg correction (FDR < 0.05)

**Table 5:** Erectile dysfunction medication use and source by occupational group (*N* = 769)

PDE5 Inhibitor Use	Digital Content Creators (n=375)	Sex Workers (n=394)	χ^2^(1)	*p*	*φ*	
Yes	179 (47.73%)	272 (69.04%)				
No	196 (52.27%)	122 (30.96%)				
*Total*	375	394	35.95	<0.000	0.22	
PDE5 Inhibitor Source	Digital Content Creators (n=179)	Sex Workers (n=272)	χ^2^(1)	*p*	*p_BH*	*φ*
Purchased Abroad	12 (6.70%)	39 (14.34%)	6.27	0.012	0.036*	0.12
Friend	44 (24.58%)	89 (32.72%)	3.44	0.064	0.096	0.09
Internet Pharmacy	32 (17.88%)	79 (29.04%)	7.26	0.007	0.036*	0.13
Online Provider (e.g. Roman, Hims)	64 (35.75%)	74 (27.21%)	3.71	0.054	0.096	0.09
Primary Care Provider	63 (35.2%)	104 (38.24%)	0.43	0.513	0.513	0.03
Other	5 (2.79%)	16 (5.88%)	2.32	0.128	0.154	0.07
Alprostadil Use	Digital Content Creators (n=375)	Sex Workers (n=394)	χ^2^(1)	*p*	*φ*	
Yes	64 (17.07%)	163 (41.37%)				
No	311 (82.93%)	231 (58.63%)				
*Total*	375	394	54.55	<0.000	0.27	
Alprostadil Source	Digital Content Creators (n=64)	Sex Workers (n=163)	χ^2^(1)	*p*	*p_BH*	*φ*
Purchased Abroad	2 (3.12%)	21 (12.88%)	4.81	0.028	0.168	0.15
Friend	30(46.88%)	64 (39.26%)	1.10	0.295	0.443	−0.07
Internet Pharmacy	11(17.19)	42 (25.77%)	1.89	0.169	0.416	0.09
Online Provider (e.g. Roman, Hims)	15 (23.44%)	52 (31.9%)	1.58	0.208	0.416	0.08
Primary Care Provider	18 (28.12%)	47 (28.83%)	0.01	0.915	0.915	0.01
Other†	0 (0.00%)	4 (2.45%)	--	0.579	0.695	0.08

† Fisher’s exact test used in place of chi-square due to zero cell; no chi-square statistic produced;

* Significant after Benjamini–Hochberg correction (FDR < 0.05)

**Table 6:** Multivariate logistic regression – Sociodemographic associations with substance use/chemsex (*N* = 733)

	aOR	95% CI	*p*
**Occupational Group**			
Digital content creator (ref)		—	—
Sex worker	3.36	2.41–4.69	<0.001***
**Age**	1.0	0.98–1.02	0.897
**Race/Ethnicity**			
White, not Hispanic or Latino (ref)		—	—
Hispanic or LatinX	0.9	0.59–1.37	0.626
Black or African American, not Hispanic or Latino	0.71	0.44–1.14	0.157
Asian, not Hispanic or Latino	0.48	0.15–1.53	0.214
American Indian or Alaska Native, not Hispanic or Latino	1.95	0.17–22.67	0.592
More than one race, not Hispanic or Latino	1.05	0.50–2.18	0.9
Other, not Hispanic or Latino	2.95	0.48–18.18	0.243
**Education**			
High school or less (ref)		—	—
Some college or associate’s degree	0.68	0.43–1.08	0.104
Bachelor’s degree	0.91	0.57–1.45	0.687
Graduate or professional degree	1.1	0.60–2.01	0.768
**Relationship Status**			
Single (ref)		—	—
Partnered	0.66	0.43–1.02	0.06
**Partner Gender**			
Any women partners (ref)		—	—
Exclusively men and/or transgender partners	0.68	0.42–1.09	0.11
**Sexual Orientation**			
Gay (ref)		—	—
Bisexual	0.49	0.28–0.84	0.010*
Other sexual minority	0.57	0.31–1.05	0.073

AOR = adjusted odds ratio; CI = confidence interval; ref = reference category. Straight/heterosexual-identifying participants (n = 11) were excluded from the regression due to small cell size and theoretical distinctiveness from sexual minority groups. Partner gender was modeled as a binary variable (exclusively men and/or transgender partners vs. any women partners) and sexual orientation was collapsed into three categories (gay, bisexual, other sexual minority) following likelihood ratio testing indicating that more granular specifications did not significantly improve model fit. Relationship status was modeled as a binary partnered/unpartnered variable for the same reason

* *p* < *0.05;*

** *p* < *0.01;*

*** *p* < *0.001*

## Data Availability

The data that support the findings of this study are not publicly available due to the sensitive nature of the information collected and the need to protect the privacy and confidentiality of the study participants. Given the potentially stigmatizing and personal nature of the data, public access could compromise participant anonymity and well-being.

## Footnotes

♦- Website information available from authors upon reasonable request.
